# Nutrient Intake and Gut Microbial Genera Changes after a 4-Week Placebo Controlled Galacto-Oligosaccharides Intervention in Young Females

**DOI:** 10.3390/nu13124384

**Published:** 2021-12-08

**Authors:** Nicola Johnstone, Susannah Dart, Paul Knytl, Arjen Nauta, Kathryn Hart, Kathrin Cohen Kadosh

**Affiliations:** 1 School of Psychology, Faculty of Health and Medical Sciences, University of Surrey, Guildford GU2 7XH, UK; s_dart@hotmail.co.uk (S.D.); p.knytl@surrey.ac.uk (P.K.); 2 FrieslandCampina, 3818 LA Amersfoort, The Netherlands; arjen.nauta@frieslandcampina.com; 3 Department of Nutritional Sciences, School of Biosciences and Medicine, Faculty of Health and Medical Sciences, University of Surrey, Guildford GU2 7XH, UK; k.hart@surrey.ac.uk

**Keywords:** GOS, intervention, gut microbiota

## Abstract

Recent interest in the gut-brain-axis has highlighted the potential of prebiotics to impact wellbeing, and to affect behavioral change in humans. In this clinical trial, we examined the impact of four-weeks daily supplementation of galacto-oligosaccharides (GOS) on self-reported nutrient intake and relationships on gut microbiota in a four-week two-armed parallel double-blind placebo controlled GOS supplement trial in young adult females. Food diaries and stool samples were collected prior to and following 28 days of supplement consumption. It was found that four weeks of GOS supplementation influenced macronutrient intake, as evident by reduced carbohydrate and sugars and increased fats intake. Further analysis showed that the reduction in carbohydrates was predicted by increasing abundances of Bifidobacterium in the GOS group in comparison to the placebo group. This suggests that Bifidobacterium increase via GOS supplementation may help improve the gut microbiota composition by altering the desire for specific types of carbohydrates and boosting Bifidobacterium availability when fiber intake is below recommended levels, without compromising appetite for fiber from food.

## 1. Introduction

Food choice is a critical factor in preventing non-communicable disease (NCD) and as such a major focus for the prevention of these diseases is reducing unhealthy and excessive food intake [1]. In particular, excessive sugar intake has been linked with increased risk of NCDs and obesity, such as free sugar added to beverages [2,3] Consequently, there is an ongoing effort on the part of governmental and health bodies to reduce free sugar intake through increased regulation and taxation of highly palatable foods, with multiple national campaigns to decrease unhealthy eating [4,5].

Stress and anxiety have long been colloquially blamed for “comfort eating”, and there is growing evidence to support the influence of stress on unhealthy eating behaviors [6,7]. As such, prebiotic supplementation may provide a way to influence unhealthy eating behaviors by down-regulating stress and anxiety. Prebiotics are non-digestible substances such as fructans and oligosaccharides found in cereals, fruits, and vegetables [8], which influence the gut-brain-axis through altering the growth or action of certain microbial genera in the gut [9]. In human and animal intervention studies, prebiotics have conferred wide-ranging benefits to neurobiological, immunological, metabolic, and behavioral processes [10]. Recent work by our group and others has shown promising effects of prebiotics on altering the trajectory of mental health outcomes by reducing anxiety and stress in humans via the gut-brain axis [11,12], and specifically psychobiotics can reduce negative attentional bias and reactivity to emotional information [11,12,13]. Dysfunction in regulation of emotional responses is seen as a major cause of anxiety [14] and similarly, it is negative emotional responses that are highlighted as the driving force behind comfort eating [15]. Evidence points towards psychobiotics’ prospective use to reduce emotional unhealthy eating. Prebiotics have also been shown to influence appetite [16]. Thus, it is possible that these effects of prebiotics have the potential to disrupt the cycle of emotional eating of highly palatable foods by decreasing both initial cravings and negative emotions.

In a recent study by our group, we highlighted the impact of dietary changes via prebiotic supplementation on the gut microbiome composition and mood and emotional behavior in a randomized placebo-control trial of the effects of a galacto-oligosaccharide (GOS) on indices of mood and well-being in young female adults [12]. This trial acquired multilevel data on nutrient intake, psychological assessments, cognitive-emotional processing, and stool sampling. Our primary analysis focused on anxiety symptomology which is disproportionally detected in females in comparison to males in this age group [17]. A single-sex sample also reduced the potential influence of other confounding factors, such as hormones. Specifically, we found that GOS supplementation over four weeks reduced trait anxieties and indicators of anxious behavior in a cognitive task for those who initially had high levels of anxiety and that these changes at the behavioral level were mirrored by significant changes in gut microbiome composition, most notably an increase in Bifidobacteria abundance [12]. In this paper, we focus on the impact of these gut microbiome changes on nutrient intake based on comprehensive food diaries that were collected for nutrient analysis.

One challenge in investigating this link is the potentially recursive relationship between diet, gut microbiota, and mood. Whereas animal feeding is opportunistic, humans have abundant choice in food selection that extends, food poverty notwithstanding, beyond the homeostatic maintenance of the body. This is important since food choice mediates commensal gut bacteria activity to influence health outcomes [18,19]. Viewed in this way, food choice is both an environmental factor in health outcomes and a behavior which responds to health outcomes [20]. Furthermore, there remains a gap in understanding how chosen foods operate on gut microbiota and, indeed, gut microbiota composition can be altered in days by dietary and environmental changes [21,22,23]. Therefore, if gut microbiota influence food choice, food choice may also influence the gut microbiome, complicating causal attribution. In addition, although pre- and probiotics have been investigated in the treatment of psychiatric disorders (e.g., anxiety, depression, neurodevelopmental disorders [24], to date there is mixed evidence for efficacy [25,26,27]. We postulate that to ascertain true prebiotic effects, data on nutritional intake must be acquired in consideration of humans as holobionts [28]. Thus, any study of the link between the gut-brain axis and changes in eating behavior must take baseline *ad libidum* eating behavior, mood, and gut microbial composition into account. We have furthermore proposed that studies of the gut microbiota in mental health must be multilevel in data acquisition [29].

To properly investigate the influence of GOS on nutrient intake and explore associations with gut microbiota, we present here our extended analysis of nutrient intake to evaluate (1) the effects of GOS (versus placebo) supplementation on macronutrients as a percentage of total energy intake in carbohydrates, fats, and protein, including sugars and free sugar, fiber, monounsaturated fatty acid and saturated fatty acids, and total calorie intake; and (2) explore associations of changes in nutrient intake with changes in gut microbiota composition in comparison to a placebo group over four-weeks.

## 2. Methods and Materials

### 2.1. Participants

Sixty-four healthy young adult female volunteers (aged 18–25 years) were recruited to a double-blind placebo-controlled four-week galacto-oligosaccharides (GOS) Biotis^TM^ GOS intervention study via posters and online advertisements. Exclusion criteria were self-reported current or previous clinical diagnoses of anxiety or co-morbid neurological, psychiatric, gastrointestinal, or endocrine disorders; current habitual use of prebiotic or probiotic supplements; antibiotic use three months prior to study enrolment, vegan diets (due to the supplement derivation from lactose sources), and BMI ≥ 30 kg/m^2^. Only females were recruited to maintain population-homogeneity in the primary outcome as trait anxiety scores tend to be higher in young student females than males [30]. Written informed consent was received from each participant prior to testing and financial compensation for participating was given. Supplement group allocation, GOS or placebo, was performed blindly using a custom program with stratification on the group median of trait anxiety scores reported on the state-trait anxiety inventory [30] to high and low anxiety groups for equitable anxiety levels across intervention groups. This study was approved by the University of Surrey Ethics Committee (UEC/2017/086/FHMS) and is registered on https://www.clinicaltrials.gov number NCT04616937 (registration date 5 May 2020). All testing and data processing were carried out in accordance with relevant guidelines and regulations.

### 2.2. Protocol

Participants completed the same testing protocol at time 1 (T1) and at time 2 (T2). T1 took place one day prior to supplement consumption commencing, and T2 four weeks later (day twenty-eight, or a close as practical). In this study, we report on food diary analysis, and associations with differentially abundant gut microbiota. Stool samples were collected at T1 and T2. A four-day estimated food diary was completed at T1 and the subsequent three days, and on four consecutive days encompassing T2 to assess average nutrient intake during these periods. In keeping the food diary, participants were explicitly instructed to not change their usual diet.

### 2.3. Materials

*Food Diaries*. Participants were instructed to note all food and drinks consumed alongside time of day with an indication of portion sizes and ingredients in recipes. Pictures provided guidance for estimating portion size. Diaries included at least one weekend day. Diaries were reviewed by a member of the research team at testing appointments and any omissions clarified and were analyzed using nutritional analysis software [31] for energy and macronutrient intakes.

*GOS/placebo supplement*. Participants received either a daily dose of 7.5 g of the prebiotic galacto-oligosaccharides (Biotis™ GOS, ~5.5 g GOS) provided by FrieslandCampina Ingredients, Amersfoort, The Netherlands; or a placebo (maltodextrin, dried glucose syrup) for a period of 28 days. GOS are non-digestible carbohydrates, which are not completely broken down by human digestive enzymes. Due to this, they reach the intestine relatively intact, where they are then available for the present microbiota, whereas maltodextrin is absorbed in the upper part of the intestine and does not reach the colon. Both supplements were provided in powdered form in unlabeled packaging and are similar in color and taste. Nutritional values of the GOS supplement were 2.9 kcal/g comprised of 0.70 g GOS; 0.22 g lactose, 0.055 g other sugars (glucose and galactose), and 0.024 g moisture; and of the placebo, maltodextrin 6.4g digestible carbohydrate 2.5 kcal/g comprised of 0.064 g glucose, 0.38 g disaccharides and 5.37 g higher polysaccharides, and 0.32 g moisture. Supplements were instructed to be consumed on consecutive days by mixing with food or drink once daily.

*Stool sampling*. At baseline and follow-up participants were provided with a unique sampling kit provided by MyMicroZoo (Leiden, the Netherlands) for stool collection at home. Feces samples were collected in DNA/RNA Shield (Zymo Research, Irvine, CA, USA) and returned by the subjects to the recruitment station and stored at −80 °C prior to being shipped on dry-ice for analysis by MyMicroZoo. DNA extraction. DNA extraction was performed using the Quick-DNA Fecal/Soil Microbe Miniprep Kit (Zymo Research) according to manufacturer’s instructions except for using the fecal slurry, containing DNA/RNA Shield, as input during bead beating for mechanical cell lysis instead of using the lysis buffer provided in the extraction kit.16S rRNA gene based bacterial profiling. Illumina 16S rRNA gene amplicon libraries were generated and sequenced at BaseClear (Leiden, The Netherlands).

### 2.4. Analysis

*Food diary analyses.* Nutrient intake was calculated as an average over four days’ food records for nine variables including total energy (in Calories) and key macronutrients expressed as a percentage of energy intake: 1. Total Carbohydrate; 2. Total fat; 3. Protein; 4. sugars (composed from free monosaccharides and disaccharides, but not oligosaccharides); 5. Free sugars (all manufacturer or participant added sugars, including in natural sources from fruit juice, honey and syrup); 6. Fiber (composed of total dietary fiber including lignin and resistant starch); 7. Saturated fatty acid; and 8. Monounsaturated fatty acid.

*Gut microbiota differential abundance testing.* Genus level reads were filtered (minimum 5000, minimal proportion of 0.01 across all samples) using the CoDaSeq R package [32,33] retaining 86 taxa for analysis. Bayesian-Multiplicative replacement of count zeros [34] was then used to impute remaining zero reads after which the center log-ratio transformation was applied to standardize taxa abundances. Differential abundance testing on each of the 86 taxa collected at T2 was performed using analysis of covariance with supplement group (GOS or placebo) as predictor and T1 taxa as covariate. Correcting for positive false discovery rate with Storey’s q-value [35] found eight taxa that differed due to intervention; *Bacteroides*, *Barnesiella*, *Gardnerella*, *Bifidobacterium*, *Aestuariispira*, *Desulfovibrio*, *Peptoniphilus* and *Sporobacter* [12]. Abundances of these genera were selected for the present analysis.

*Statistics.* Analysis of covariance models (ANCOVAs) were used to model intervention effects (GOS or Placebo) on the nutrient variables independently, expressed as a percentage of energy. T1 measures of each outcome were covaried in addition to body-mass-index (BMI) recorded at T1. The residuals of each ANCOVA were evaluated for normality with Shapiro-Wilks test. Where this was significant q-q plots were inspected for outliers, tested using a Bonferroni outlier test [36] and Cook’s distance for influential cases on model fit. If significant outliers were found, these were removed and the ANCOVA repeated.

To explore further intervention effects on nutrient variables, we used stepwise selection regression in both forward and backwards directions [37] to model gut microbiome bacteria noted as differentially abundant in Johnstone et al. (2021) for establishing if intervention-induced effects on the gut microbiome predict dietary changes. Stepwise regression begins with a fully defined model of possible predictors and sequentially removes each to evaluate effects on model fit, before adding back in predictors and re-evaluating model fit. ‘Best fit’ predictors are retained to explain contribution to observed outcomes, in this case, predictors are the eight microbiome genera observed as differentially abundant between groups at T2, and outcomes are nutrient variables.

The predictive bacteria selected were *Bacteroides*, *Barnesiella*, *Gardnerella*, *Bifidobacterium*, *Aestuariispira*, *Desulfovibrio*, *Peptoniphilus* and *Sporobacter*. Delta values (T2-T1 change in bacterial abundance, expressed as a percentage of total gut microbiome composition) were used for nutrient outcomes and bacteria predictors, with T1 measured BMI included as a covariate. The residuals from significant models were assessed for normality using Shapiro-Wilks test. All analyses were carried out in R version 4.0.2 [38]; data were managed using base functions, dplyr (version 1.0.6, [39]) and broom (version 0.7.6, [40]), and statistics calculated using the base packages stats in addition to car (version 3.0-10, [36]), jtools (version 2.13, [41]), and MASS (version 7.3-51.6, [37]). Images were produced using ggplot2 (version 3.3.3, [42]). Effects are significant at *p* < 0.05.

## 3. Results

Of the 64 participants enrolled on the study, 16 did not return food diaries at time 1 (10 assigned to the GOS treatment group and 6 assigned to the placebo group). Of the remaining 48 participants, a further 2 participants did not return T2 measures with the remaining sample of 46 completing the supplement intervention and T2 measures, 23 in the GOS treatment group (age *M* = 19.97 years, *SD* = 1.85; BMI *M* = 21.94 kg/m^2^ *SD* = 3.39), and 23 in the placebo group (age *M* = 20.07 years, *SD* = 1.70; BMI *M* = 20.84 kg/m^2^ *SD* = 2.67).

### 3.1. Intervention Effects on Nutrient Outcomes

Prior to running ANCOVAs, all data were assessed for baseline difference in treatment groups, with BMI as a covariate. Energy intake was found to be greater in the placebo group (*M* = 1943.64 kcal *SE* = 67.46) compared to the GOS group (*M* = 1692.73 kcal, *SE* = 70.46) at T1 (*F*(1,45) = 5.42, *p* = 0.024). No further measures differed, confirming the suitability of ANCOVA.

**Figure 1:**
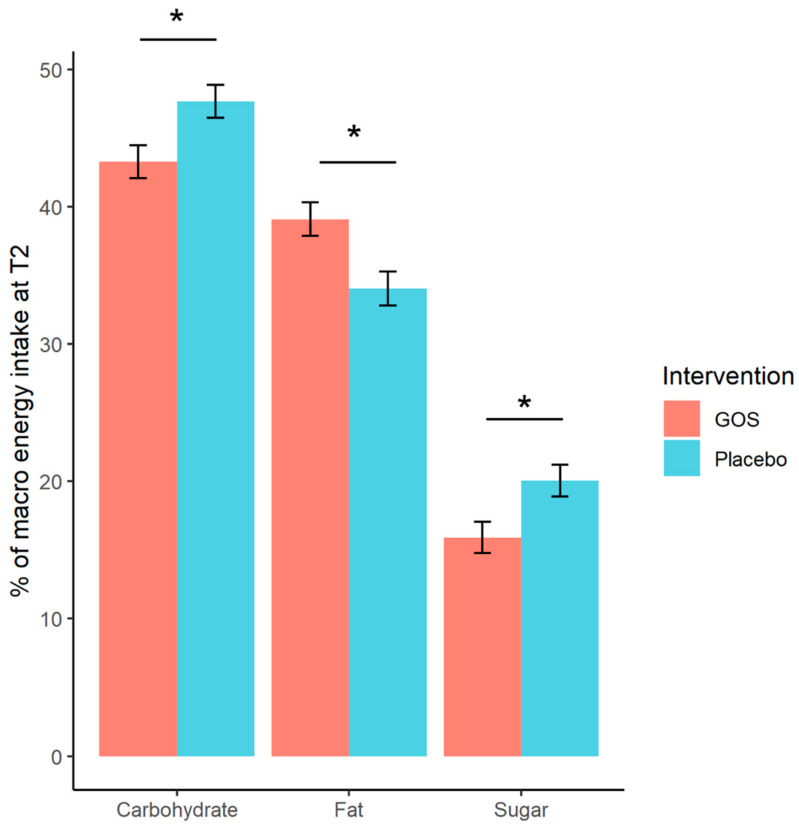
Significant treatment effects from ANCOVA tests on nutrient intake at T2 for carbohydrates, fat, and sugars (*n* = 23 in each group). The GOS intervention shows reduced total carbohydrate and reduced sugars compared to the placebo group and increased total fat intake. Error bars are standard error of the mean. * *p* < 0.05.

Descriptive means of nutrient intake in each group at T1 and T2 are displayed in Table 1, and additionally in Supplementary Table S1. Evaluating treatment effects with ANCOVAs at T2 (including T1 response and BMI as covariates) found significantly reduced carbohydrates in the GOS group and reduced sugars intake compared to placebo. One high-leverage outlier was removed from the analysis of sugar outcome (this case presented an atypical recording pattern suggestive of under-reporting at T2 with a reduction of 23.5% in sugars intake from T1 to T2), model residuals thereafter followed a normal distribution. Fat was additionally found to significantly increase after GOS treatment in comparison to placebo group. These significant effects are illustrated in Figure 1. There were no treatment effects on protein, fiber or free sugars, or monounsaturated fatty acid or saturated fatty acid. The full ANCOVA model reports for each outcome can be found in Supplementary Table S2.

### 3.2. Exploring Intervention Effects on Gut Microbiota in Predicting Nutritional Intake

Descriptive statistics for significantly differential abundances of eight genera between treatment groups (reported in Johnstone et al., 2021) are displayed in Table 2, these genera are *Bacteroides*, *Barnesiella*, *Gardnerella*, *Bifidobacterium*, *Aestuariispira*, *Desulfovibrio*, *Peptoniphilus* and *Sporobacter*. Group level delta abundances were entered into stepwise regression models as predictors for each delta nutrient intake (total carbohydrates, total fats, monounsaturated fatty acid, saturated fatty acid, protein, fiber, sugars, and free sugars), with each outcome assessed in separate models. T1 BMI was entered as a covariate.

The stepwise regression for carbohydrates was found to be significant when BMI, *Bifidobacterium*, *Barnesiella* and *Desulfovibrio* were included, of these GOS *Bifidobacterium* was a negative predictor and GOS *Desulfovibrio* a positive predictor in carbohydrate change, with normal distribution of residuals. This means that in the GOS group increasing *Bifidobacteria* and decreasing *Desulfovibrio* abundances predicts decreasing carbohydrate energy intake.

The stepwise regression for fiber was also significant only when *Bifidobacterium* was included. Placebo *Bifidobacterium* was a negative predictor of fiber change where GOS *Bifidobacterium* was not a significant predictor, with normally distributed residuals. Here, increasing abundances of *Bifidobacterium* in the placebo group predicted reduced fiber energy intake.

Protein change was significantly predicted by a model including only *Bifidobacterium* in which both the placebo group was a negative predictor and the GOS group a positive predictor. Residuals were normally distributed. These results illustrate that energy intake from protein are predicted by reduced *Bifidobacterium* in the placebo group and increased *Bifidobacterium* in the GOS group.

The model for free sugar change was significant when *Bifidobacterium*, *Peptoniphilus* and *Sporobacter* were included. Placebo *Bifidobacterium* was a positive predictor and placebo *Peptoniphilus* also a positive predictor. Model residuals were normally distributed. This indicates that only in the placebo group was increasing abundances of *Bifidobacterium* and *Peptoniphilus* predictive of increased energy from free sugars.

Saturated fatty acid change predicted by a model including only *Peptoniphilus,* in which the placebo group was a was negative predictor. Distribution of model residuals were normal. Herein increasing *Peptoniphilus* abundances predict increased energy from saturated fatty acids.

**Figure 2:**
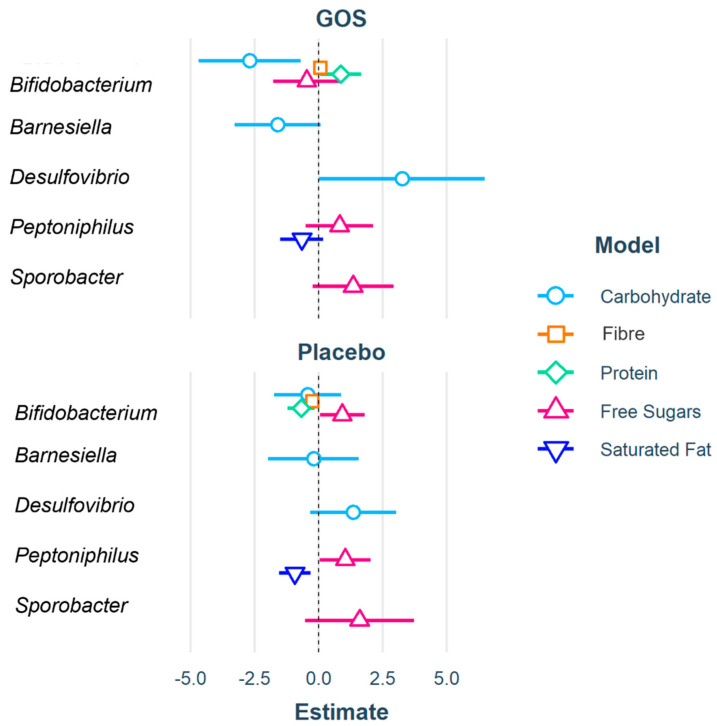
Coefficients from significant stepwise regressions for each nutrient model (carbohydrate, fibre, protein, free sugars, and saturated fat), represented by different colors and point shapes. Only genera which had a significant contribution on model outcomes are plotted. Treatment groups (GOS and Placebo) are plotted separately for ease of viewing. The further the data point from central zero the larger the coefficient and strength of effect. Data points to the left of zero are negative coefficients, illustrating a negative relationship of the predictor to nutrient outcome, and data point to the right are positive coefficients, representing a positive relationship. In the carbohydrate model (blue), GOS *Bifidobacterium* and *Desulfovibrio* are significant coefficients. In the Fibre model (orange), placebo *Bifidobacterium* is a significant coefficient. The Protein model (green) found GOS *Bifidobacterium* a positive significant coefficient and placebo *Bifidobacterium* a negative significant coefficient. The free sugar model (pink) found placebo *Bifidobacterium* and *Peptoniphilus* to be significant coefficients and in the saturated fat model (dark blue) placebo *Peptoniphilus* a negative significant coefficient.

These significant models are displayed in Table 3, and the coefficients depicted in Figure 2. Full model results are reported in Supplementary Table S3. The stepwise regression models for total fat, monounsaturated fatty acid and sugar changes were unrelated to any of the included predictors.

## 4. Discussion

Previous research has highlighted the role of prebiotic supplementation in influencing appetite [16] and the potential to disrupt the cycle of emotional eating of excessive unhealthy foods, via decreasing anxiety [11,15]. Here we show that GOS supplementation over four weeks altered nutrient intake in healthy females with 4.3% less energy from carbohydrate, 4.2% more energy from fats, and 4.1% less energy from sugar in comparison to a placebo group. Linking nutrient intakes to differential abundances of specific bacteria in an exploratory analysis found that the reduction in carbohydrates is driven by increased *Bifidobacterium* following GOS supplementation. There was no similar effect of *Bifidobacterium* on total or free sugars, suggesting increasing abundances of *Bifidobacterium* influence nutrient intakes from digestible fibers, although this warrants further fine-grained analysis.

Fat intake increased, and sugar intake decreased, when expressed as a proportion of total energy intake, in the GOS group in comparison to the placebo group, although we found no associations of these effects in the gut microbiota. Further, there was no evident change in fiber intake between supplement groups, so it may be inferred that GOS supplementation does not replace dietary fiber intake, nor have a direct influence on total fat and total sugar consumption. It may be that GOS has a specificity mechanism in increasing abundance of Bifidobacterium that reduces the need to eat specific types of carbohydrates. Carbohydrates are a principal energy source of diverse components, from monosaccharides to complex polysaccharides [43]. A recent meta-analysis found carbohydrate composition, e.g., complex polysaccharides opposed to monosaccharides to be important in reducing the risk of non-communicable disease in the general population and specifically highlighted dietary fiber and whole grains as key targets for improving and protecting health outcomes [44]. However, focusing on the consumption of specific fibers as actors of health benefits (e.g., the ‘prebiotic effect’) may be misguided as argued by Makki and colleagues (2018), and rather, the functional and ecological effects of fiber fermentation (e.g., short-chain fatty acid [SCFA] production) may be more relevant for health outcomes.

There are substantial and differential effects of fibre supplementation on gut microbiota [45], as enhanced fibre consumption increases specific gut taxa, but changes are individualized [45,46,47] and the consequences of this effect SCFA production. SCFAs such as acetate, propionate, and butyrate are end product metabolites produced by bacterial fermentation of prebiotics in the colon. SCFAs modulate host health (including gut barrier function, glucose homeostasis, immunomodulation, appetite regulation and obesity) and microbial activity in the gut [48,49,50]. SCFA are natural ligands and considered important for signaling between gut microbes and the host [51]. Although precise mechanisms in humans are still under investigation, it is thought that as signaling molecules in host metabolism, SCFAs bind to nutrient sensing free fatty acid receptors (FFARs) which respond flexibly to changes in nutritional state altering immune and metabolic response as directed [52]. In this way, nutrient intake drives host health via nutrient uptake in gut microbiota composition.

Where fiber intake is crucial to supporting the gut microbiota in SCFA production and host response [53], protein fermentation may by be used to produce SCFAs when fiber is in short supply. Protein fermentation increases branch chain fatty acids (BCFA) and alters the production of metabolic compounds potentiating increased inflammation and disease in the gut [54] again highlighting nutritional status as a driver of host health. We postulate that while human homeostasis is highly adaptive, it is subject to behavioral actions in food selection that has a strong influence on overall health via gut microbiome composition.

Obtaining sufficient fiber intake through food choice is not a hallmark of western diets and may be responsible for increasing rates of metabolic and cardiovascular disease [55]. Typical daily fiber consumption is around 19g, where 30g is recommended (~3% of energy intake) [46]. GOS may therefore be useful to plug the fiber gap. Our participants consumed around 2% of energy from fiber on average. However, those in the GOS group had 11.5% greater *Bifidobacterium* abundance compared to the placebo group. *Bifidobacterium* is an intestinal bacterium supporting the production of SCFA by increasing availability of polysaccharides to other commensal bacteria [56], an adaptive resource that may protect SCFA production where fiber is lacking in the diet.

Food choice to support nutrient intake is a complex topic involving individual, societal, and environmental factors. Using food diaries to measure nutrient intake over several days provides a snapshot of a typical diet but can lack objectivity [57] and have inconsistencies with observed eating behavior change [16], and so including additional food intake measures may give a clearer account of nutrient changes. To help unravel some of the complexity in these data, it would be particularly beneficial to include food frequency estimates in order to elucidate which foods on the plate are driving the nutrient changes observed, and if GOS supplementation complements a change in specific types of food chosen. Still, here we have shown that gut bacterial composition exerts an influence.

We showed that in the group receiving GOS supplementation increasing *Bifidobacterium* abundance was related to reduced carbohydrate intake and increasing *Desulfovibrio* to increased carbohydrate intake. *Desulfovibrio* bacteria are generally associated with clinical intestinal disease [58], although certain species have been found to beneficial in non-alcoholic fatty acid liver disease [59]. The compositional abundances of *Desulfovibrio* in these data are low, and occurrence in the GOS group were greater than in the placebo group. However, under GOS supplementation, *Desulfovibrio* bacteria abundance typically reduce with *Bifidobacterium* growth [60,61]. It is reasonable to conclude that the regression of Desulfovibrio in the carbohydrate effects are influenced by two cases that increased more than 1.5 standard deviations than group average. Yet, it is important to stress that gut microbiota is compositional in nature, and the relative abundances of one genus in reference to all others is important in determining health and effects. While the mechanisms of single genera and derived species can be elucidated, this must be considered in refence to all genera present, particularly in otherwise healthy populations.

We also found reduced energy from sugar, and increased energy from fats that were not associated with gut bacterial composition. Baseline levels of energy from fats in the GOS group initially were above recommended levels and only increased, and no reductions in energy from free sugars were evident. This may be a less than optimal diet profile, depending on specific foods consumed, and highlights the limits of unidimensional dietary interventions for affecting health outcomes, and is a limitation of this study where appetite is not measured. However, several other studies have illustrated that food interventions (typically of fibers) can change food choice behaviors [62,63] and appetite [64]. Nevertheless, integrating multiple levels of analysis from bacteria functions, genes, and metabolite production to understand the complexity in appetite and cravings might be better served by metabolomic approaches [65]. This is promising for designing individualized nutrient interventions to improve host health. While making food choices is complex, identifying strategies that will lead to better well-being would assist in making healthier decisions–advice that is desirable particularly for young people, who are still undergoing significant developmental changes [25].

This exploratory study found that four weeks of GOS supplementation influenced nutrient intake in relation to carbohydrate, fats, and sugar. Further, the reduction in carbohydrates was predicted by increasing abundance of *Bifidobacterium* in the GOS group. This suggests that *Bifidobacterium* increase via GOS supplementation may help improve the gut composition by altering the desire for specific types of carbohydrates and boosting *Bifidobacterium* availability when fiber intake is below recommended levels, without compromising appetite for fiber from food.

## Figures and Tables

**Table 1:** Descriptive measures of nutrient intake pre- and post-intervention for young women, by treatment group.

	GOS (*n* = 23)						
Measure	T1 *M*	(*SD*)	T2 *M*	(*SD)*	∆*M*	(*SD*)	
Energy (Kcal)	1631.68	(338.09)	1556.04	(501.17)	−102.20	(376.35)	↓
Protein (%E)	16.18	(4.54)	16.62	(4.09)	0.29	(2.77)	↑
Fat (%E)	36.05	(6.54)	39.31	(7.28)	3.63 *^B^	(6.40)	↑
Monounsaturated fatty acid (%E)	11.55	3.29	13.47	5.25	1.63	5.16	↑
Saturated fatty acid (%E)	12.58	(3.40)	13.40	(3.41)	1.11	(2.84)	↑
Carbohydrate (%E)	45.28	(7.12)	42.60	(7.58)	−2.77 *^A^	(5.82)	↓
Free Sugars (%E)	8.75	(5.48)	9.23	(5.25)	0.11	(1.42)	↑
Sugars (%E)	18.85	(7.61)	16.25	(4.93)	−3.21 *^A^	(5.62)	↓
Fibre (%E)	2.26	(0.64)	2.20	(0.69)	−0.04	(0.17)	↓
	**Placebo (*n* = 23)**						
	T1 *M*	(*SD*)	T2 *M*	(*SD*)	∆*M*	(*SD*)	
Energy (Kcal)	1921.62	(418.33)	1724.03	(452.41)	−212.47 *^A^	(367.84)	↓
Protein (%E)	15.35	(4.33)	16.10	(4.45)	0.74	(2.87)	↑
Fat (%E)	35.01	(6.04)	33.81	(4.89)	−1.06	(7.56)	↓
Monounsaturated fatty acid (%E)	12.11	3.35	11.64	2.50	−0.64	3.76	↓
Saturated fatty acid (%E)	11.65	3.14	11.91	3.27	0.38	3.13	↑
Carbohydrate (%E)	47.19	(6.41)	48.36	(6.47)	1.13	(6.54)	↑
Free Sugars (%E)	8.45	(4.40)	9.02	(4.77)	0.30	(1.44)	↑
Sugars (%E)	18.95	(6.79)	19.27	(8.20)	0.17	(7.93)	↑
Fibre (%E)	1.95	(0.65)	1.99	(0.61)	0.01	(0.33)	↑

Note. Average (mean, *M*) nutrient intakes as a percentage of energy (%E) at T1 and T2, and difference (T2 minus T1) with standard deviations (*SD*) presented for each group separately. Significance tests evaluating the change across time were calculated and are denoted by asterisks; * *p* < 0.05. Letters denote paired sample test used; ^A^ significance value from two-sided paired sample T-test statistic for gaussian response distributions, ^B^ significance value from Wilcoxon signed rank test for non-gaussian response distributions, identified by significant Shapiro-Wilk test of normality across groups. GOS = galacto-oliogosaccharides. Arrows are illustrative indications of change direction ↑ increase, ↓ decrease.

**Table 2:** Descriptive measures of influential genera expressed as a percentage of total gut bacteria counts pre and post intervention in young women, by treatment group.

Measure	T1 *M*	(*SD*)	T2 *M*	(*SD*)	∆*M*	(*SD*)	
	**GOS *n* = 21**						
*Aestuariispira*	−3.30	(1.54)	−2.77	(2.35)	0.54	(1.82)	↑
*Bacteroides*	5.69	(1.13)	5.49	(1.53)	−0.20	(0.71)	↓
*Barnesiella*	1.23	(2.39)	1.82	(2.25)	0.59	(1.51)	↑
*Bifidobacterium*	3.82	(1.96)	4.62	(1.37)	0.80 **^B^	(1.28)	↑
*Desulfovibrio*	−1.45	(2.30)	−1.30	(2.57)	0.15	(0.87)	↑
*Gardnerella*	−3.83	(0.65)	−3.64	(0.75)	0.18	(0.59)	↑
*Peptoniphilus*	−3.32	(1.10)	−3.36	(1.21)	−0.04	(1.41)	↓
*Sporobacter*	0.11	(1.75)	0.48	(1.72)	0.37	(1.15)	↑
	**Placebo *n* = 23**						
*Aestuariispira*	−3.30	(2.04)	−3.59	(1.26)	−0.32	(1.40)	↓
*Bacteroides*	5.11	(1.15)	5.30	(1.23)	0.24	(0.59)	↑
*Barnesiella*	1.59	(2.06)	1.19	(2.17)	−0.32 *^B^	(1.58)	↓
*Bifidobacterium*	3.93	(1.88)	4.14	(2.18)	0.01	(2.05)	↑
*Desulfovibrio*	−1.72	(2.44)	−2.34	(2.25)	−0.56	(2.00)	↓
*Gardnerella*	−3.73	(1.15)	−2.97	(1.59)	0.75 **^B^	(1.03)	↑
*Peptoniphilus*	−3.24	(1.47)	−2.50	(1.63)	0.70	(1.90)	↑
*Sporobacter*	0.48	(1.72)	0.23	(2.11)	−0.17	(0.89)	↓

Note. Average (mean, *M*) abundance of significant bacteria as a percentage of total gut bacteria composition (%) at T1 and T2, and difference (T2 minus T1) with standard deviations (*SD*) presented for each group separately. Significance tests evaluating the change across time were calculated and are denoted by asterisks; * *p* < 0.05, ** *p* < 0.01. Letters denote paired sample test used; ^B^ significance value from Wilcoxon signed rank test for non-gaussian response distributions, identified by significant Shapiro-Wilk test of normality across groups. GOS = galacto-oliogosaccharides. Arrows are illustrative indications of change direction ↑ increase, ↓ decrease.

**Table 3:** Stepwise regression results for each nutrient model.

	Carbohydrate	Fibre	Protein	Free Sugar	Saturated Fat
(Intercept)	12.20	0.08	0.04	−0.79	1.10 *
	[−2.04, 26.44]	[−0.09, 0.25]	[−0.80, 0.88]	[−2.22, 0.63]	[0.26, 1.94]
BMI	−0.52				
**GOS**	[−1.17, 0.13]				
*Bifidobacterium*	−2.70 **	0.07	0.86 *	−0.47	
	[−4.69, −0.71]	[−0.10, 0.23]	[0.06, 1.65]	[−1.78, 0.84]	
*Barnesiella*	−1.60				
	[−3.27, 0.08]				
*Desulfovibrio*	3.26 *				
	[0.02, 6.49]				
*Peptoniphilus*				0.82	−0.66
				[−0.51, 2.14]	[−1.50, 0.19]
*Sporobacter*				1.34	
				[−0.23, 2.92]	
**Placebo**					
*Bifidobacterium*	−0.43	−0.25 ***	−0.68 *	0.92 *	
	[−1.74, 0.89]	[−0.36, −0.14]	[−1.22, −0.15]	[0.05, 1.79]	
*Barnesiella*	−0.20				
	[−1.97, 1.57]				
*Desulfovibrio*	1.35				
	[−0.33, 3.04]				
*Peptoniphilus*				1.04 *	−0.93 **
				[0.04, 2.04]	[−1.54, −0.32]
*Sporobacter*				1.60	
				[−0.53, 3.72]	
N	44	44	44	44	44
*R* ^2^	0.34	0.34	0.22	0.31	0.23

Beta coefficients are reported with 95% confidence intervals in brackets. Significant effects are denoted: *** *p* < 0.001; ** *p* < 0.01; * *p* < 0.05. Sample size is given by N, and variance explained by contributing predictors on nutrient outcomes is given by *R*^2^.

## Data Availability

Data supporting these results are stored on OSF here: https://osf.io/e5ymk/?view_only=d196d94319044644a241820f9f7ed4f3, accessed on 2 December 2021.

## Supplementary Materials

The following are available online at https://www.mdpi.com/article/10.3390/nu13124384/s1. Table S1: Descriptive measures of nutrient intake in recorded units (grams) pre- and post intervention for young women, by treatment group; Table S2: ANCOVA model statistics of intervention effects on nutrient outcomes; Table S3: Stepwise regression models of gut microbiota on each nutrient outcome.
