# Supplementary material for: Nutrient Intake and Gut Microbial Genera Changes after a 4-Week Placebo Controlled Galacto-Oligosaccharides Intervention in Young Females
Source: Nutrients. 2021 Dec 8;13(12):4384. doi: 10.3390/nu13124384 (PMC8705328; doi:10.3390/nu13124384)
Supplement: Supplementary file 1 [file nutrients-13-04384-s001.zip › nutrients-1472117-supplementary.pdf]

Nutrient intake and gut microbial genera changes after a 4-week placebo controlled galacto-oligosaccharides intervention in young females

Table S1. Descriptive measures of nutrient intake in recorded units (grams) pre- and post intervention for young women, by treatment group.

	T1 *M*	(SD)	T2 *M*	(SD)	Δ*M*	(SD)	
**GOS n = 23**							
**Protein**	65.88	35.17	62.32	32.64	-5.03	13.13	↓
**Fat**	65.68	34.38	67.63	37.53	1.28	16.19	↑
Monounsaturated fat	20.83	11.22	22.46	13.28	0.96	7.62	↑
Saturated Fat	23.12	12.98	23.62	14.06	0.45	6.61	↑
**Carbohydrate**	184.79	94.33	167.67	95.85	-20.36	40.09	↓
Free Sugars	36.53	27.31	28.29	20.11	-9.82	21.58	↓
Sugars	78.96	48.65	65.34	41.18	-16.54	27.34	↓
Fibre	18.37	9.92	17.82	10.47	-0.70	4.24	↓
**Placebo n = 23**							
**Protein**	73.59	38.62	67.83	34.82	-6.33	13.53	↓
**Fat**	75.66	37.50	65.42	33.93	-10.60	21.79	↓
Monounsaturated fat	26.18	14.06	22.21	11.28	-4.46	9.67	↓
Saturated Fat	25.30	13.92	22.96	13.40	-2.34	6.94	↓
Carbohydrate	225.18	102.34	208.41	105.04	-18.61	42.06	↓
**Free Sugars**	40.52	25.96	38.93	31.17	-3.00	20.54	↓
Sugars	89.53	48.38	84.85	55.62	-5.91	23.06	↓
Fibre	18.57	9.86	18.23	9.63	-0.72	6.26	↓

Note. Average (mean, *M*) nutrient intakes in grams at T1 and T2, and difference (T2 minus T1) with standard deviations (*SD*) presented for each group separately. GOS = galacto-oligosaccharides. Arrows are illustrative indications of change direction ↑ increase, ↓ decrease.

Table S2. ANCOVA model statistics of intervention effects on nutrient outcomes

			Model results			Estimated marginal means		
Measure	term	df	*F*	*p*	η2		*M*	*SE*
**Carbohydrate**	T1	1	29.193	0	0.368			
	BMI	1	1.621	0.21	0.02			
	**Treatment**	**1**	**6.546**	**0.014**	**0.082**	**GOS**	43.28%	1.19
	Residuals	42				**Placebo**	47.66%	1.19
**Sugars**	T1	1	13.755	0.001	0.233			
	BMI	1	0.281	0.599	0.005			
	**Treatment**	**1**	**6.41**	**0.015**	**0.09**	**GOS**	15.90%	1.13
	Residuals	42				**Placebo**	20.05%	1.16
**Fat**	T1	1	7.94	0.007	0.135			
	BMI	1	0.489	0.488	0.008			
	**Treatment**	**1**	**8.278**	**0.006**	**0.141**	**GOS**	39.10%	1.23
	Residuals	42				**Placebo**	34.05%	1.23
MUF	T1	1	2.525	0.12	0.053			
	BMI	1	0.486	0.49	0.01			
	Treatment	1	2.468	0.124	0.052			
	Residuals	42						
SFA	T1	1	22.646	0	0.342			
	BMI	1	0.182	0.672	0.003			
	Treatment	1	1.407	0.242	0.021			
	Residuals	42						
Protein	T1	1	72.313	0	0.632			
	BMI	1	0.027	0.87	0			
	Treatment	1	0.098	0.756	0.001			
	Residuals	42						
Fiber	T1	1	24.465	0	0.365			
	BMI	1	0.509	0.48	0.008			
	Treatment	1	0.046	0.832	0.001			
	Residuals	42						
Free Sugars	T1	1	7.984	0.007	0.154			
	BMI	1	0.073	0.788	0.001			
	Treatment	1	1.87	0.179	0.036			
	Residuals	42						
Energy Kcal.	T1	1	31.885	0	0.427			
	BMI	1	0.486	0.49	0.007			
	Treatment	1	0.28	0.599	0.004			
	Residuals	42						

Note. Treatment effect was comparison of GOS group in reference to the placebo group at T2. Outcome measure collected at time 1 and body mass index were included as covariates. Distribution of residuals for significant models were all normal; carbohydrate, $w = 0.981$, $p = .666$; sugar, $w = 0.952$ $p = .063$ and fat, $w = 0.96$, $p = .120$.

Table S3. Stepwise regression models of gut microbiota on each nutrient outcome

	β	2.5%	97.5%	t	p	Partial r	Part r
**Carbohydrate model**							
(Intercept)	12.200	-2.041	26.441	1.737	0.091		
BMI	-0.521	-1.172	0.131	-1.620	0.114	-0.261	-0.220
Bifidobacterium: Treatment Placebo	-0.426	-1.738	0.886	-0.658	0.515	-0.109	-0.089
***Bifidobacterium*: Treatment Active**	**-2.696**	**-4.686**	**-0.705**	**-2.747**	**0.009**	-0.416	-0.373
Treatment Placebo: *Barnesiella*	-0.201	-1.970	1.568	-0.231	0.819	-0.038	-0.031
Treatment Active: *Barnesiella*	-1.598	-3.274	0.077	-1.934	0.061	-0.307	-0.263
Treatment Placebo: *Desulfovibrio*	1.353	-0.331	3.036	1.630	0.112	0.262	0.221
**Treatment Active: *Desulfovibrio***	**3.257**	**0.024**	**6.489**	**2.043**	**0.048**	0.322	0.277
Model fit $F(7,36) = 2.603, p = 0.028, R^2 = 0.336$							
Distribution of residuals: $W = 0.977$ $p = 0.505$							
**Fiber model**							
(Intercept)	0.078	-0.094	0.251	0.920	0.363		
***Bifidobacterium*: Treatment Placebo**	**-0.247**	**-0.357**	**-0.137**	**-4.543**	**0.000**	-0.579	-0.576
***Bifidobacterium*: Treatment Active**	**0.066**	**-0.098**	**0.230**	**0.811**	**0.422**	0.126	0.103
Model fit $F(2,41) = 10.64, p < 0.001, R^2 = 0.342$							
Distribution of residuals: $W = 0.980$ $p = 0.808$							
**Protein model**							
(Intercept)	0.042	-0.797	0.881	0.101	0.920		
***Bifidobacterium*: Treatment Placebo**	**-0.681**	**-1.217**	**-0.146**	**-2.569**	**0.014**	-0.372	-0.355
***Bifidobacterium*: Treatment Active**	**0.855**	**0.057**	**1.653**	**2.162**	**0.036**	0.320	0.299
Model fit $F(2,41) = 5.63, p = 0.007, R^2 = 0.216$							
Distribution of residuals: $W = 0.99$ $p = 0.963$							
**Free sugar model**							
(Intercept)	-0.794	-2.221	0.633	-1.128	0.267		
Bifidobacterium: Treatment Placebo	0.921	0.053	1.790	2.149	0.038	0.333	0.293
Bifidobacterium: Treatment Active	-0.470	-1.778	0.837	-0.729	0.471	-0.119	-0.099
**Treatment Placebo: *Peptoniphilus***	**1.037**	**0.036**	**2.038**	**2.099**	**0.043**	0.326	0.287
Treatment Active: *Peptoniphilus*	0.815	-0.508	2.138	1.248	0.220	0.201	0.170
Treatment Placebo: *Sporobacter*	1.596	-0.530	3.723	1.521	0.137	0.243	0.208
Treatment Active: *Sporobacter*	1.342	-0.235	2.920	1.725	0.093	0.273	0.235
Model fit $F(6,37) = 2.77, p = 0.025, R^2 = 0.310$							
Distribution of residuals: $W = 0.95$ $p = 0.053$							
**Saturated fatty acid model**							
(Intercept)	1.102	0.264	1.940	2.656	0.011		
**Treatment Placebo: *Peptoniphilus***	**-0.927**	**-1.537**	**-0.317**	**-3.071**	**0.004**	-0.432	-0.422
Treatment Active: *Peptoniphilus*	-0.656	-1.497	0.186	-1.573	0.123	-0.239	-0.216
Model fit $F(2,41) = 5.975, p = 0.005, R^2 = 0.226$							
Distribution of residuals: $W = 0.980$ $p = 0.770$							
